# *Peromyscus leucopus*, *Mus musculus*, and humans have distinct transcriptomic responses to larval *Ixodes scapularis* bites

**DOI:** 10.1128/iai.00065-25

**Published:** 2025-03-11

**Authors:** Jeffrey S. Bourgeois, Julie E. McCarthy, Siu-Ping Turk, Stephanie S. You, Quentin Bernard, Luke H. Clendenen, Gary P. Wormser, Luis A. Marcos, Kenneth Dardick, Sam R. Telford, Adriana R. Marques, Linden T. Hu

**Affiliations:** 1 Tufts Lyme Disease Initiative, Department of Molecular Biology and Microbiology, Tufts University School of Medicine 12261 , Boston, Massachusetts, USA; 2 Laboratory of Clinical Immunology and Microbiology, National Institute of Allergy and Infectious Diseases (NIAID), NIH 573242 , Bethesda, Maryland, USA; 3 Division of Infectious Diseases, Department of Medicine, New York Medical College 8137 , Valhalla, New York, USA; 4 Department of Medicine, Division of Infectious Diseases, Department of Microbiology and Immunology, Stony Brook University 214388 , Stony Brook, New York, USA; 5 Mansfield Family Practice 161852 , Storrs, Connecticut, USA; 6 Tufts Lyme Disease Initiative, Department of Infectious Disease and Global Health, Tufts University 1810 , North Grafton, Massachusetts, USA; Washington State University, Pullman, Washington, USA

**Keywords:** Lyme disease, *Ixodes scapularis*, *Peromyscus leucopus*, *Mus musculus*, human, transcriptomics, immune response, wound healing, tick bites

## Abstract

*Ixodes scapularis* ticks are an important vector for at least seven tick-borne human pathogens, including a North American Lyme disease spirochete, *Borrelia burgdorferi*. The ability for these ticks to survive in nature is credited, in part, to their ability to feed on a variety of hosts without triggering an immune response capable of preventing tick feeding. While the ability of nymphal ticks to feed on a variety of hosts has been well documented, the host-parasite interactions between larval *I. scapularis* and different vertebrate hosts are relatively unexplored. Here we report on the changes in the vertebrate host transcriptome present at the larval tick bite site using the natural *I. scapularis* host *Peromyscus leucopus*, a non-natural rodent host, *Mus musculus* (BALB/c), and humans. We note substantially less evidence of activation of canonical proinflammatory pathways in *P. leucopus* compared to BALB/c mice and pronounced evidence of inflammation in humans. Pathway enrichment analyses revealed a particularly strong signature of interferon gamma, tumor necrosis factor, and interleukin 1 signaling at the BALB/c and human tick bite sites. We also note that bite sites on BALB/c mice and humans, but not deer mice, show activation of wound-healing pathways. These data provide molecular evidence of the coevolution between larval *I. scapularis* and *P. leucopus* and, in addition, expand our overall understanding of *I. scapularis* feeding.

## INTRODUCTION

*Ixodes scapularis* (formerly *Ixodes dammini*) ticks are the most important invertebrate vector of human diseases in North America (1). These ticks are responsible for spreading most cases of Lyme disease (predominantly caused by *Borrelia burgdorferi sensu stricto* in North America), which affects an estimated 476,000 individuals in the United States yearly (2), as well as six other human pathogens—*Anaplasma phagocytophilum*, *Babesia microti*, *Borrelia miyamotoi*, *Borrelia mayonii*, *Ehrlichia muris eauclairensis*, and deer tick virus/Powassan virus (3, 4). Tick feeding can trigger immunologic processes at the bite site in the skin, which may threaten the ability of the tick to survive during the blood meal (5). To prevent this from occurring, *Ixodes* ticks secrete saliva containing anticoagulants and immunomodulatory compounds into the feeding site, which dampens the host’s immune response and ensures that the tick can remain attached until completion of feeding (6–8). This secretion also contributes to the spread of *B. burgdorferi* into new hosts, both by providing a mechanism to exit the tick (9–11) and by dampening the immune response while the pathogen establishes the infection (12–19).

*I. scapularis* is a generalist parasite that successfully feeds on many hosts (20). In line with this, previous work has demonstrated that nymphal tick bites on guinea pigs (*Cavia porcellus*), *Mus musculus*, *Peromyscus leucopus*, and humans are broadly similar at the histopathological level (21–23). However, major changes across species occur during recurrent tick bites, where, for instance, guinea pigs and humans develop a greater level of skin inflammation than other animal species with repeat exposure, resulting in itching, rejection of the tick, and/or reduced risk of pathogen transmission (21, 23, 24). Notably, at the transcriptional level, differences between the *M. musculus* (BALB/c) and *C. porcellus* immune responses to nymphal *I. scapularis* were apparent during the first feeding (23). This demonstrates that not all potential *I. scapularis* hosts are equally permissive to parasitization by the tick.

Relatively little attention has been paid to how any vertebrate—including humans or rodents—interact with larval *I. scapularis*, although field studies have documented a strong association between larvae and *P. leucopus* in the northeastern and midwestern United States (20, 25–27), as well as with birds and reptile hosts in the southeastern United States (20, 28, 29). In his 1989 review, Ribeiro stated that their unpublished data demonstrated that larval *I. scapularis* can feed efficiently on the North American deer mouse *P. leucopus* (also called the white-footed mouse) but not on *C. porcellus* (5). Similarly, larval *I. scapularis* were found to feed better on *P. leucopus* than *Microtus pennsylvanicus* voles (30). While larval *I. scapularis* can be infected with some tick-borne pathogens (e.g*.*, *Babesia* spp. [31] and *Borrelia miyamotoi* [32]), they are not infected with *B. burgdorferi* (33) and less commonly bite humans compared with the nymphal stage of this tick species (34); thus, they have limited direct clinical impact on patient health compared to later tick stages. However, this stage does have a major indirect impact on Lyme disease by impacting the *B. burgdorferi* infection abundance in nature as larval tick feeding is critical for the continuation of the *B. burgdorferi* enzootic cycle (1).

In this study, we compare the transcriptomic response of two rodent models of tick feeding, the natural *I. scapularis* host *P. leucopus* and an inbred *M. musculus* strain (BALB/c), to examine the transcriptomic response to larval *I. scapularis*. Pathway enrichment analyses revealed activation of more proinflammatory signaling pathways in *M. musculus* than in *P. leucopus*. We also note evidence of tissue remodeling and homeostasis genes activating in BALB/c rodents but not *P. leucopus*. Integration of a previously published transcriptomic data set examining the nymphal bite site in BALB/c mice demonstrated considerable differences between the *M. musculus* immune response to each of these tick stages, suggesting that ticks may have different capacities to suppress proinflammatory pathways in different hosts across the stages in the tick life cycle. We also evaluated human patient samples (35) to compare how the human transcriptional response compared to these rodent responses. We found a strong proinflammatory immune response to larval *I. scapularis* tick bites in humans, with activation of many of the same pathways (tumor necrosis factor [TNF], interferon gamma [IFN-γ], and interleukin 1 [IL-1] signaling) as in *M. musculus*. Here we provide a deeper look at the differences between mammalian species in response to larval *Ixodes* tick bites.

## RESULTS

### BALB/c mice and *Peromyscus leucopus* deer mice respond differently to larval *Ixodes scapularis*

**Fig 1:**
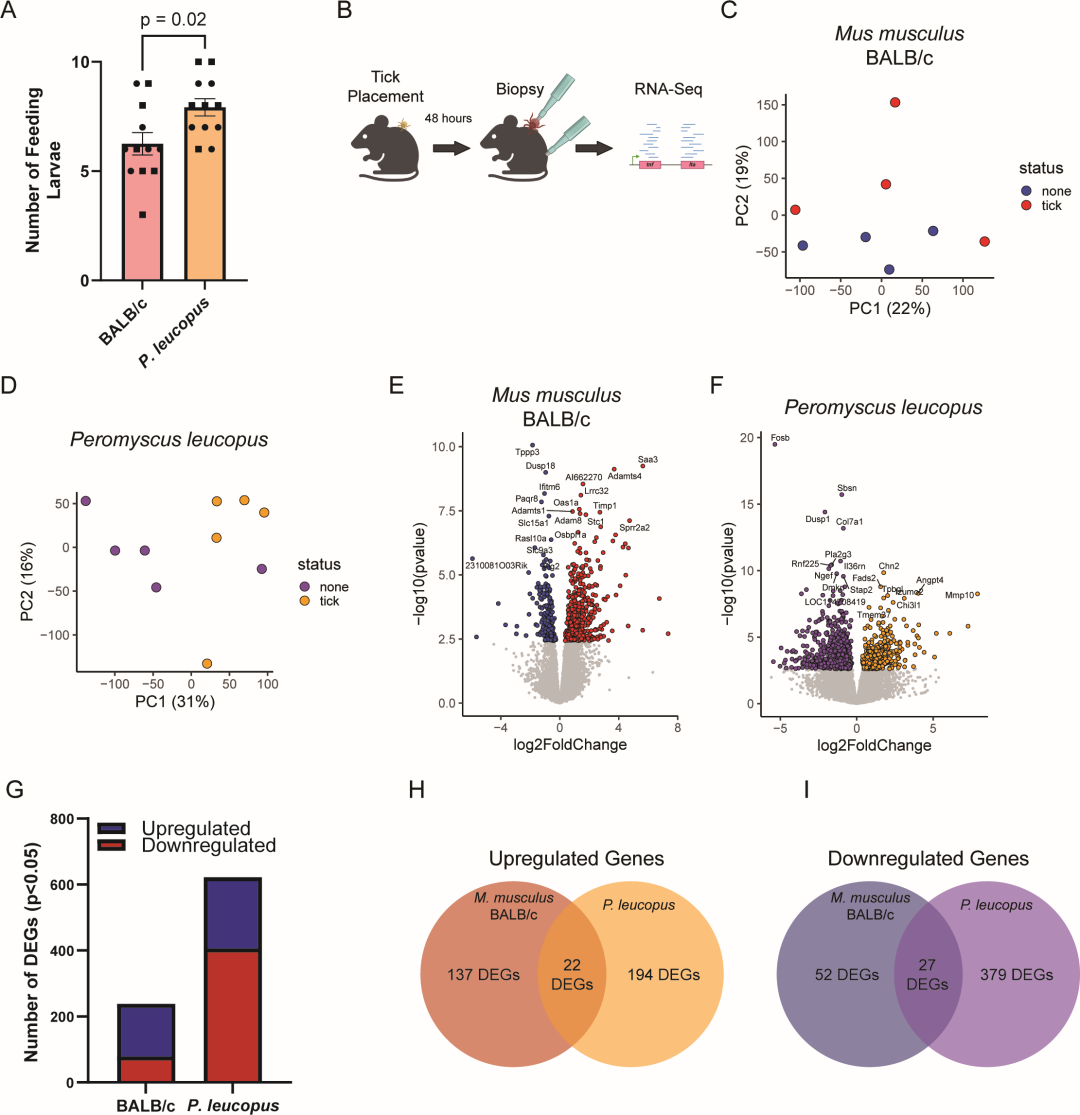
BALB/c *M. musculus* and *P. leucopus* display different transcriptomic responses to bites from larval *I. scapularis*. (**A**) Ten larval *I. scapularis* were placed under placement caps on rodents, and the number of attached, feeding larvae were quantified at 48 hours post-placement. Each dot represents a single rodent. Squares represent male rodents; circles represent females. *P* value from Mann-Whitney test. Data from four independent experiments. (**B**) Schematic of larval tick placement and RNA-seq. (**C and D**) Principal component analysis based on observed transcripts per million reads for each gene in (**C**) BALB/c mice or (**D**) *P. leucopus*. (**E and F**) Volcano plots of differentially expressed genes in (**E**) BALB/c mice or (**F**) *P. leucopus*. Genes with *P* > 0.05 are shown in gray. (**G**) BALB/c mice display more upregulation of genes than downregulation, while *P. leucopus* spp. display more downregulation of genes than upregulation. (**H and I**) Most genes (**H**) upregulated or (**I**) downregulated in BALB/c or *P. leucopus* are not similarly differentially expressed in the opposite rodent.

We first examined whether there are differences in the ability for larval *I. scapularis* to feed on BALB/c and *P*. leucopus. We placed 10 larval ticks in a tick containment chamber affixed to the back of each rodent. Then, the number of attached and feeding ticks 48 hours post-placement was assessed, roughly one-half to three-quarters of the way through feeding. We noted a small but consistent increase in the number of feeding ticks attached to *P. leucopus* at 48 hours compared to BALB/c rodents (Fig. 1A). In order to examine how BALB/c (*n* = 4) and *P. leucopus* (*n* = 5) rodents respond to larval *I. scapularis* bites, 2 mm punch biopsies were taken at the site of a feeding tick at 48 hours post-placement. Additionally, a second punch biopsy was taken from a region outside of the tick containment chamber as a control (Fig. 1B).

We performed RNA sequencing on these biopsies. Principal component analysis (PCA) revealed that tick bite sites cluster away from unbitten skin along the second principal component in BALB/c mice (Fig. 1C) and along the first principal component in *P. leucopus* (Fig. 1D). We next performed differential gene expression analysis (Table S1) to examine genes that were induced or suppressed (*P*_adj_ <0.05) by larval tick feeding. For all analyses, gene lists were restricted to those genes that could be reliably identified across all samples, meaning across different skin biopsy sites (tick bite and control) and across the rodent species (BALB/c and *P. leucopus*). Differential gene expression analyses revealed that 238 genes were differentially expressed in BALB/c tick bite sites (Fig. 1E), and 622 genes were differentially expressed in *P. leucopus* tick bite sites (Fig. 1F). Examining the direction of effect, we found that most differentially expressed genes in BALB/c were upregulated (159 upregulated and 79 downregulated), while most genes in *P. leucopus* were downregulated (216 upregulated and 406 downregulated) (Fig. 1G). There was remarkably little overlap between the upregulated genes or downregulated genes between BALB/c mice and *P. leucopus* (Fig. 1H and I).

To check whether the use of a tick containment chamber could affect gene responses, we performed control experiments in an independent group of mice comparing biopsies under the chamber and outside the chamber without a tick bite. We used reverse transcription quantitative PCR (RT-qPCR) to quantify changes in three of the most highly differentially regulated genes between *P. leucopus* and *M. musculus* related to inflammation or wound healing (*Ccr5*, *Ccr7*, and *Fosb*). We found that the chamber did not alter *Ccr5* expression in either species, drove a small increase in *Ccr7*, and reduced expression of *Fosb* in BALB/c rodents but not in *P. leucopus* (Fig. S1A). We concluded that while the chamber may cause changes to some transcripts, the impact of the chamber is minimal and likely within normal variation.

We attempted to replicate some cross-species differences we observed in our RNA sequencing data set (*Ccl5*, *Ccr7*, and *Fosb*; Fig. S1B) using eight additional rodents from each species. While we were not able to replicate the trends we saw in *Ccl5* and *Ccr7* expression, we were able to replicate our observation that *Fosb* is significantly downregulated in *P. leucopus* but not in BALB/c (Fig. S1C). Of note, these changes in expression did not correlate with differences in the number of ticks that successfully fed (Fig. S1D). Therefore, we conclude that our RNA sequencing experiment was able to identify species-dependent responses to larval *I. scapularis*, but do note that follow-up studies are required to confirm any individual association.

### Ingenuity Pathway Analysis reveals more proinflammatory cytokine signaling in BALB/c mice compared to *P. leucopus* at tick bite sites

The QIAGEN Ingenuity Pathway Analysis (IPA) pipeline (36) can be used to identify signaling pathways that show evidence of activation or suppression in response to the tick bite. This program assigns pathways controlled by a given upstream regulator (e.g*.*, cytokines and transcription factors). Importantly, this is based on expression of genes across the entire pathways. Thus, these values cannot be interpreted as meaning that the upstream regulator itself (e.g*.*, *Tnf*) is upregulated or downregulated in each data set.

**Fig 2:**
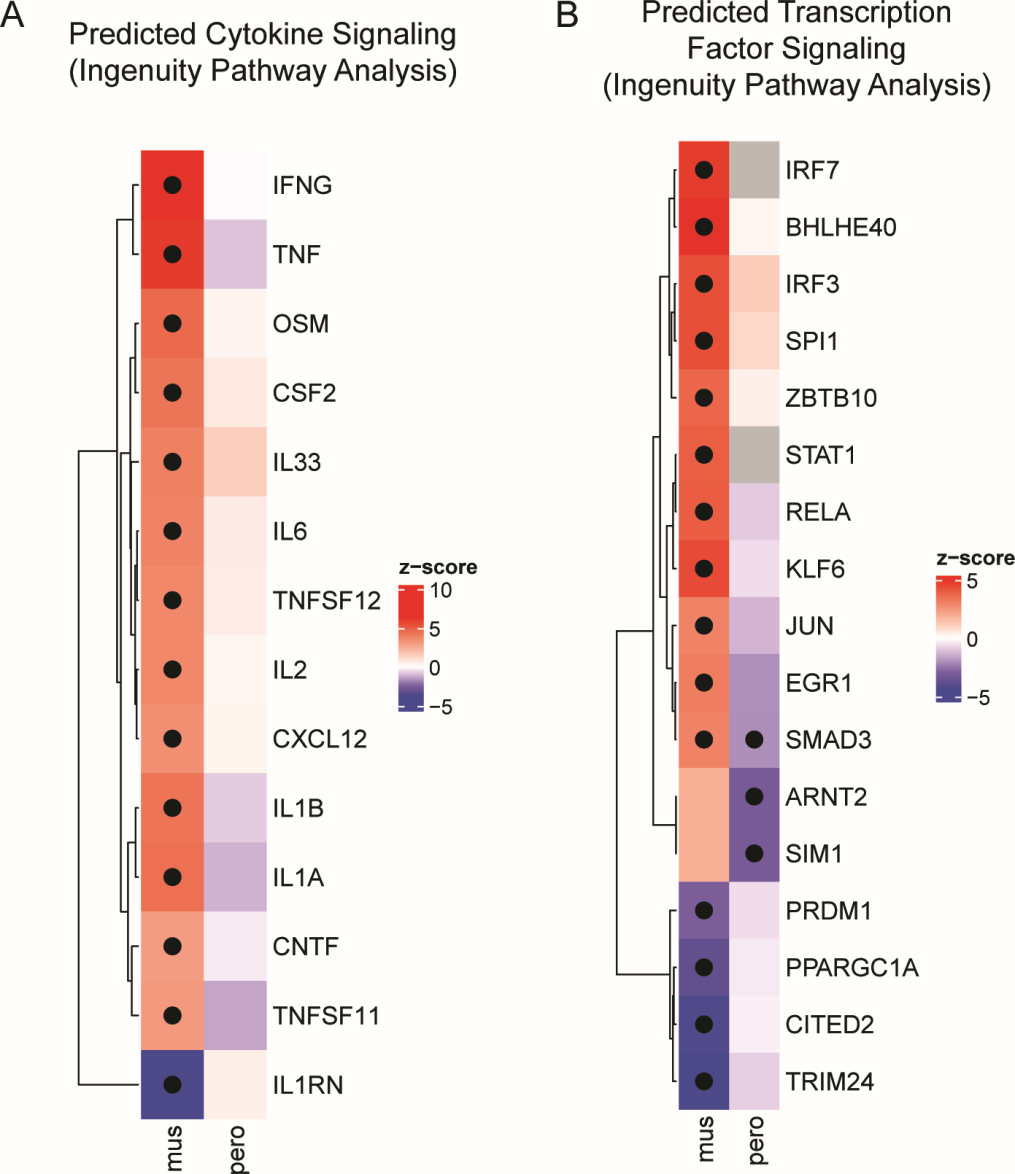
Ingenuity Pathway Analysis reveals differences in predicted pathway activation. (**A**) Cytokine-regulated pathways identified by QIAGEN IPA software (36) that were predicted to be differentially activated (*Z*-score greater than or equal to 3 or less than or equal to −3) in either BALB/c mice or *P. leucopus* and had a *Z*-score difference of 2 or greater between BALB/c and *P. leucopus*. (**B**) Transcription factor-regulated pathways identified by QIAGEN IPA software (36) that were predicted to be differentially activated (*Z*-score greater than or equal to 3 or less than or equal to −3) in either BALB/c mice or *P. leucopus* and had a *Z*-score difference of 2 or greater between BALB/c and *P. leucopus*. For panels A and B, color represents direction of effect (red denotes increased signaling; blue denotes reduced signaling); dots represent a significant *P* value for pathway enrichment (*P* < 0.05).

Using this tool, we identified 48 cytokine-regulated pathways that showed evidence (*Z*-score ≥2) of activation and 1 pathway that showed evidence (*Z*-score ≤−2) of suppression in BALB/c mice (Table S2). In contrast, three cytokine-regulated pathways showed evidence of activation and two showed evidence of suppression in *P. leucopus* (Table S2). Of these, two pathways (IFNB1 and PRL) were activated in both species. We focused our attention on the 23 cytokine-regulated pathways that had (i) the most significant predicted activation (*Z*-score >3) or repression (*Z*-score <−3) in at least one of the species and (ii) at least a *Z*-score difference of 2 between the two species (Fig. 2A). This revealed multiple proinflammatory pathways that were predicted to have substantial activation in BALB/c mice but not *P. leucopus*, particularly the TNF-regulated (BALB/c *Z*-score 6.132, *P. leucopus Z*-score −0.867) and IFN-γ-regulated pathways (BALB/c *Z*-score 6.4, *P. leucopus Z*-score −0.02). We also noted increased predicted IL-6 and IL-1 signaling in BALB/c mice but not *P. leucopus*. Conversely, BALB/c mice had substantive predicted repression of the anti-inflammatory interleukin 1 receptor antagonist (IL1RN)-regulated pathway, which showed no evidence of regulation in *P. leucopus* (BALB/c *Z*-score −3.633, *P. leucopus Z*-score 0.454). Together, these data suggest that the BALB/c mice show signs of robust proinflammatory cytokine signaling at the transcriptomic level that is substantially less present in *P. leucopus*.

### Ingenuity Pathway Analysis reveals differences in activity of transcription factor-regulated pathways in BALB/c mice and *P. leucopus* bite sites

IPA revealed 41 transcription factor-regulated pathways that showed evidence of activation (*Z*-score ≥2) in BALB/c tick bite sites and 19 transcription factor-regulated pathways with evidence of repression (*Z*-score ≤−2) (Table S2). There were 4 transcription factor-regulated pathways that showed evidence of enhanced activity in *P. leucopus* and 17 that showed evidence of repression. Of these, two transcription factor-regulated pathways (ETS1 and GATA2) were predicted to be activated in both rodents.

Similar to our cytokine-regulated pathway analyses, we focused our attention on transcription factor-regulated pathways, where we observed (i) the most significant predicted activation (*Z*-score >3) or repression (*Z*-score <−3) in at least one of the rodent species, and (ii) at least a *Z*-score difference of 2 between the two species, which includes 37 pathways (Fig. 2B). Complementing our cytokine data, we noted that numerous proinflammatory pathways were predicted to be activated in BALB/c but not in *P. leucopus*, including IRF3 (BALB/c *Z*-score 4.35, *P. leucopus Z*-score 1.182) and RELA (BALB/c *Z*-score 4.04, *P. leucopus Z*-score −0.898). STAT1 (BALB/c *Z*-score 4.001) and IRF7 (BALB/c *Z*-score 4.851) pathways were predicted to be activated in BALB/c, but a *Z*-score could not be calculated in the *P. leucopus* data set. Wound healing pathways, including the JUN-regulated pathway, were also predicted to be activated in BALB/c but not *P. leucopus* (BALB/c *Z*-score 3.062, *P. leucopus Z*-score −1.292), which is notable as the AP-1 transcription factor is suppressed during nymphal tick feeding on *M. musculus* (37). Other cell proliferation and wound healing pathways also appear active specifically in BALB/c, including EGR1 (BALB/c *Z*-score 3.221, *P. leucopus Z*-score −1.993) and SMAD3 (BALB/c *Z*-score 3.072, *P. leucopus Z*-score −2.028). Transcription factor-regulated pathways that were predicted to be suppressed in BALB/c mice but unaffected in *P. leucopus* included the HIF1A-repressor CITED2 (BALB/c *Z*-score −4.27, *P. leucopus Z*-score −0.282) and TRIM24 (BALB/c *Z*-score −4.33, *P. leucopus Z*-score −0.816).

### Comparison of larval tick bite transcriptomics with past examinations of nymphal tick bites

**Fig 3:**
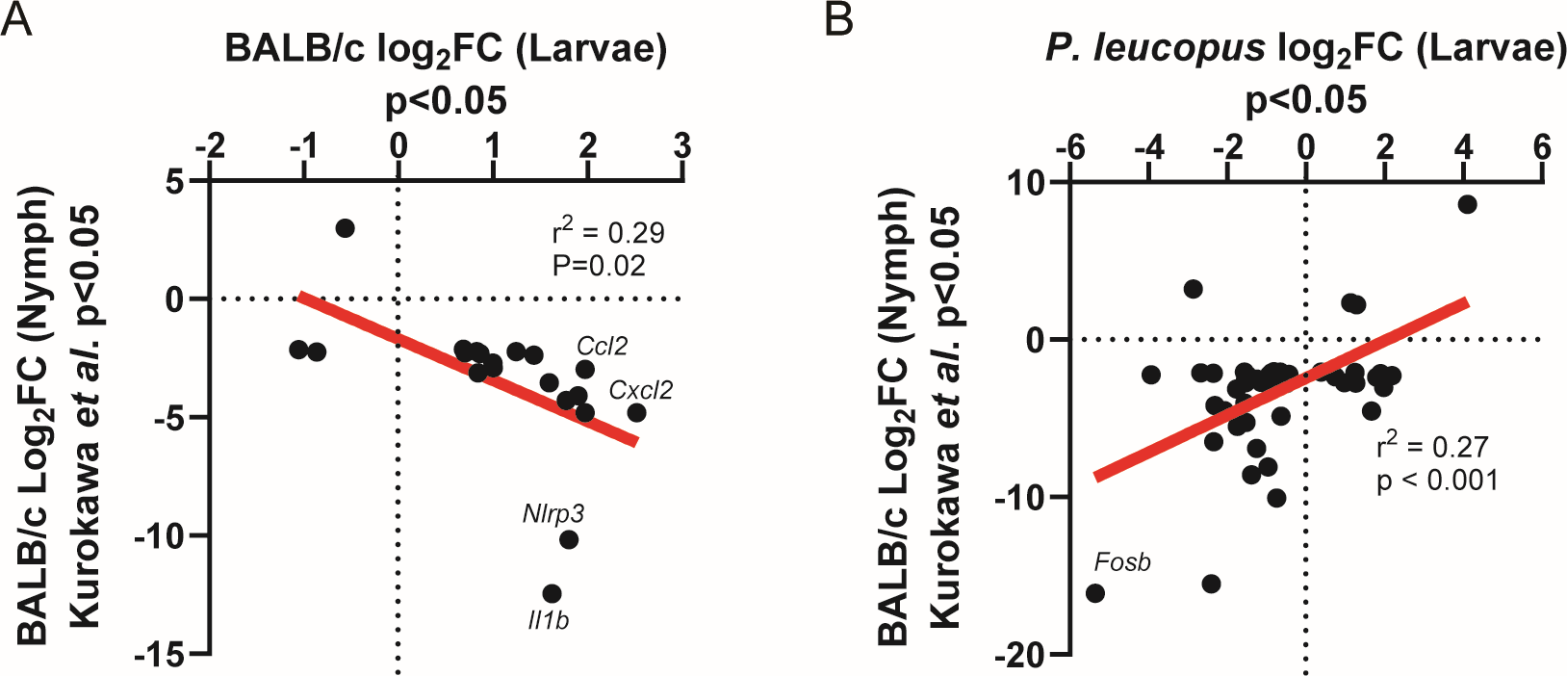
BALB/c mice, but not *P. leucopus*, display substantially different gene expression patterns following a larval tick bite compared to a BALB/c nymphal tick bite. (**A and B**) Comparison of differentially expressed genes (*P* < 0.05) in the (**A**) BALB/c or (**B**) *P. leucopus* larval tick bite site compared to differentially expressed genes in the BALB/c nymphal tick bite site reported by Kurokawa et al. (23). Statistics derived from simple linear regression and *P* value describe a slope deviation from 0.

Work by Kurokawa et al. examined transcriptomic changes in BALB/c skin following a nymphal stage *I. scapularis* tick bite (23). We compared our data from larval tick bites with these transcriptomic data from nymphal bite sites. Of note, this examined the transcriptome at a later time point (4 days post-placement) than we used here (2 days post-placement). Even considering this caveat, we were surprised that when we examined genes that were reported as differentially expressed in BALB/c in both studies, we saw a negative correlation (*r*^2^ = 0.29, *P* = 0.02), with most shared differentially expressed genes that are downregulated in Kurokawa et al. being upregulated at the larval bite site (Fig. 3A). Notably, this includes proinflammatory factors *Il1b*, *Nlrp3*, *Ccl2*, and *Cxcl2*. In contrast, when comparing the *P. leucopus* larval bite site differentially expressed genes to the BALB/c nymphal bite site differentially expressed genes, a positive correlation was observed (*r*^2^ = 0.27, *P* < 0.001): most genes were downregulated in both bite sites (Fig. 3B). For instance, the AP-1 subunit *Fosb* was downregulated at both the nymphal BALB/c bite site and the larval *P. leucopus* bite site. Together, these results demonstrate that while we observed proinflammatory signatures in the BALB/c larval tick bite site, the BALB/c nymphal tick bite site and the *P. leucopus* larval bite site appeared to be more consistently anti-inflammatory.

### Human larval tick bites show macroscopic and transcriptomic evidence of inflammation

Together, our data suggested that larval *I. scapularis* may be better suited at suppressing proinflammatory pathways in a native host (*P. leucopus*) than in a non-native host (*M. musculus*). This led us to hypothesize that as hosts become more evolutionarily divergent from *P. leucopus*, *I. scapularis* larvae will become less able to suppress inflammation, even on hosts where nymphs are well described as being able to feed. To test this hypothesis, we took advantage of a human trial where patients with prior Lyme disease who had completed antibiotic therapy were exposed to 25–30 laboratory-reared larval ticks. Skin biopsies were taken before tick placement and/or after ticks were allowed to feed to repletion (NCT02446626) (Table 1). Unlike our rodent hosts, we routinely noted strong, macroscopic observations of inflammation at the human site of larval tick feeding (Table 1), though this may have been driven in part by the fact that these patients had previously been exposed to ticks in nature (24).

**TABLE 1:** Demographics of sequenced subjects

Subject code	Analysis group (feeding)	Age	Sex	No. of tick bites (past 5 years)	Total no. fed engorged	Total no. partially fed	Total ticks	Local reaction (definitely or probably related to the tick bite)
A001^*a*^	Good feeding	70–79	Male	5	17	4	21	None
A002	Good feeding	70–79	Female	1	17	4	21	Mild pruritus
A003	Good feeding	60–69	Female	0	16	4	20	Mild pruritus
A004	Good feeding	30–39	Male	2	21	2	23	None
A005	Good feeding	50–59	Male	1	20	8	28	Mild pruritus
A006	N/A^*c*^	60–69	Male	5	15	2	17	None
A007	N/A^*c*^	50–59	Male	1	12	3	15	None
A009	Good feeding	60–69	Female	0	12	11	23	None
B001^*a*^	Bad feeding	60–69	Female	2	3	1	4	Mild pruritus and erythema
B002	Bad feeding	70–79	Male	2	4	3	7	Mild pruritus and moderate tenderness
B003^*a*^	Bad feeding	50–59	Male	2	2	4	6	Mild pruritus
B004	Bad feeding	20–29	Female	10	2	1	3	Mild pruritus
B005^*b*^	Bad feeding	50–59	Male	0	2	7	9	Mild pruritus, pain, and tenderness
B006^*b*^	N/A^*c*^	50–59	Male	0	10	3	13	Mild pruritus and tenderness
B007^*a*^	N/A^*c*^	50–59	Male	3	7	0	7	Vesicle at site
B008	N/A^*c*^	50–59	Male	1	4	10	14	Mild pruritus
B009	Bad feeding	40–49	Male	1	0	2	2	None
B010	Bad feeding	60–69	Female	3	3	4	7	Mild pruritus

^*a*^ These subjects had two tick placements; this information represents the first removals for B001, B004, and B007 and second removals for A001 and B003.

^*b*^ B005 and B006 are the same subject. B006 is the second tick placement/removal.

^*c*^ Samples with >10 but <20 recovered fed ticks were not included for good vs bad feeding analyses.

We performed RNA sequencing on biopsies that met one of the following criteria: (i) there was a biopsy taken prior to tick placement and after tick removal (pre-tick placement vs post-tick placement); (ii) the post-tick removal biopsy was collected from a subject who had at least 20 total fed ticks (good feeding); or (iii) the post-tick removal biopsy was collected from a subject who had less than 10 total fed ticks with less than half of those being fully fed ticks (bad feeding). There were 29 skin biopsy samples that met these criteria and were processed for RNA sequencing from 17 participants, with one subject having biopsies sequenced from two xenodiagnostic procedures (Table 1).

Prior to discussing our results, we note three important caveats for comparing these data to our rodent data set. First, different sources of ticks were used between the studies. In order to reduce the risk of transovarially passed tick-borne pathogens, ticks used for human feeding were derived from a closed laboratory colony. Second, human biopsies were taken at the end of tick feeding rather than at an intermediate time point as was used in our rodent studies. Third, unlike our rodents, some humans had prior tick exposure. While these caveats may drive some of the differences we observed, we note that this is the first examination of larval tick bites in any of these species and thus present these data as a launching point for future studies.

**Fig 4:**
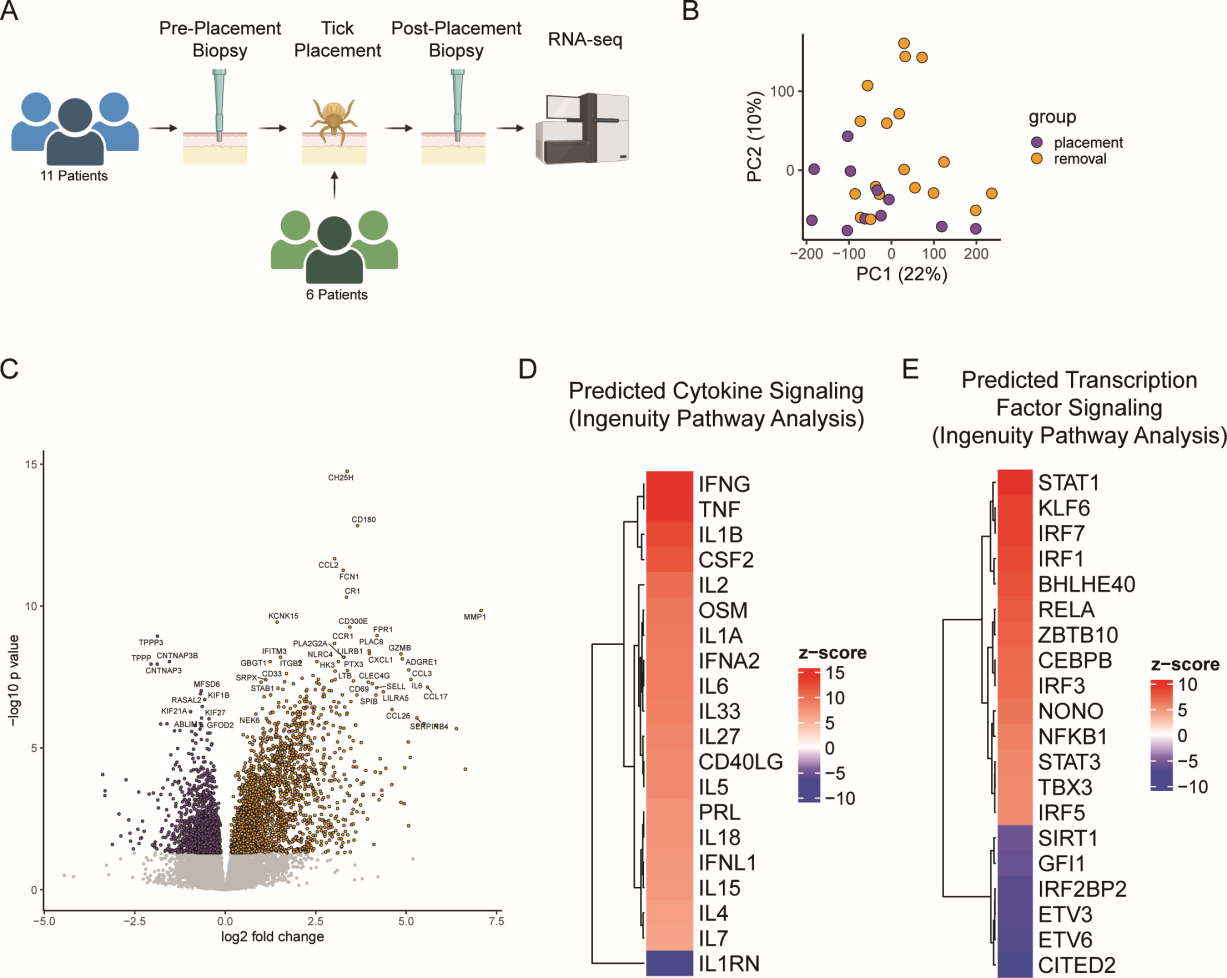
Transcriptomic analysis of the human larval *I. scapularis* bite site. (**A**) Schematic of patient sample collection. (**B**) Principal component analysis based on observed transcripts per million reads for each gene in humans. (**C**) Volcano plot displaying differentially expressed genes in response to the larval tick bite. Genes with *P* > 0.05 are displayed in gray. (**D and E**) IPA analysis of the top 20 predicted differentially activated pathways regulated by (**D**) cytokines or (**E**) transcription factors following a larval tick bite in humans. All listed upstream regulators have a *Z*-score greater than or equal to 2 or less than or equal to −2.

Skin biopsies were collected from a subset of patients prior to tick placement and all patients after tick removal to examine the effects on the transcriptional profile of human skin after larval tick bites (Fig. 4A). Principal component analysis revealed samples from bitten skin modestly clustered along the second principal component (Fig. 4B). Differential gene expression analysis (*P*_adj_ <0.05) revealed 4,322 differentially expressed genes (Fig. 4C; Table S3), 2,686 of which were upregulated and 1,636 were downregulated.

While the differences in study design discussed above make direct comparisons difficult, we do note that this degree of differential expression and the overall effect sizes observed are substantially larger in humans than in rodents, which align with our hypothesis that the response to *I. scapularis* larvae is less blunted than in natural hosts. Furthermore, we noted robust evidence of inflammation, including significant upregulation of the macrophage chemoattractants CCL2, CCL3, and CCL4; the monocyte chemoattractant CCL8; the neutrophil chemoattractants CXCL1, CXCL2, and CXCL8; and the proinflammatory cytokines IL-6, IL-1B, TNF, and IL-32. We noted upregulation of CD14 and CD68, markers of dendritic cells and macrophages, respectively. The adaptive immune system also appears to play a role, with B lymphocyte attractant CXCL13, T-cell markers CD4 and CD69, and T-cell attractants CXCL9, CXCL10, CXCL11, and CCL2 all significantly upregulated in the post-tick removal samples.

Using IPA analysis, we observed an overall pattern of elevated proinflammatory cytokine signaling in bitten human skin, including activation of TNF, IFN-γ, IL-1, STAT3, STAT1, and NfKb pathways, among others (Table S4; Fig. 4D and E). We also observed suppression of IL1RN and CITED2 pathways. Notably, these pathways largely overlap with what we observed with BALB/c, suggesting that the BALB/c response to larval *I. scapularis* may be more similar to the human response than to the *P. leucopus* response—although the overall evidence of inflammation in humans (both macroscopically and in the transcriptomic data set) was substantially stronger. Recent work has examined the human transcriptome in response to a nymphal *I. scapularis* tick bite (38) and observed upregulation of IL-17-mediated inflammation, which we also observed in this data set.

### Comparison of transcriptional profiles at tick bite sites between patients with high vs low percentage of tick feeding identifies a small set of differentially expressed genes

**Fig 5:**
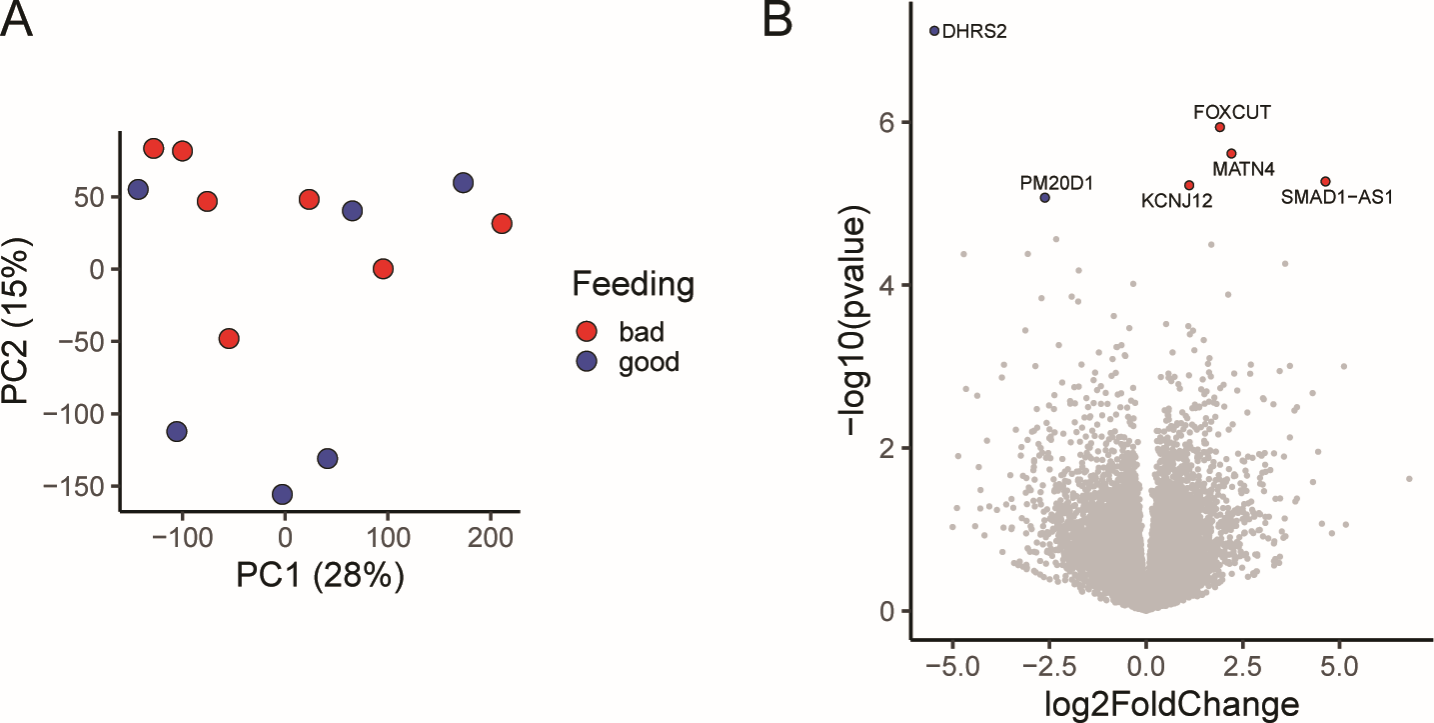
There are limited transcriptional differences between humans who had good and bad larval tick feeding. (**A**) Principal component analysis fails to separate individuals who had good feeding (≥20 fed ticks) or bad feeding (≤10 fed ticks) based on observed transcripts per million reads for each gene. (**B**) Volcano plot displaying differentially expressed genes in individuals that had good or bad feeding. Bad feeding was used as the reference data set for differential expression. Genes with *P* > 0.05 are displayed in gray.

Our observations that human patients had different abilities to support larval tick feeding led us to question if there were differences in transcriptional profiles which may be responsible for “good” (≥20 fed ticks recovered) or “bad” feeding (≤10 fed ticks recovered). Good and bad feeding did not correlate with the number of reported tick exposures in the last 5 years (*P* = 0.2, linear regression), pruritus (*P* = 0.4, *t*-test), or sex (*P* = 0.9, *t*-test). We compared the gene expression profiles of biopsies taken at the tick removal visit; the PCA of normalized gene expression data showed no distinct separation between good and bad feeding along the first and second principal components (Fig. 5A). Differential expression analysis revealed only six differentially expressed genes between feeding groups, with bad feeding as the reference group (Fig. 5B; Table S3). There were two genes, DHRS2 and PM20D1, which were upregulated with a *P*_adj_ value cutoff of 0.05 and four genes, FOXCUT, MATN4, KCNJ12, and SMAD1-AS1, which were downregulated with a *P*_adj_ cutoff of 0.05. None of these differentially expressed genes have previously been implicated in response to tick feeding and are not associated with an immune response.

## DISCUSSION

In this study, we observed divergent transcriptomic responses to bites from larval *I. scapularis* by *P. leucopus* compared with *M. musculus*. We specifically noted that *P. leucopus* display less evidence of skin inflammation based on the transcriptome than *M. musculus*, particularly when using pathway analysis software to estimate the overall evidence of inflammation. Overall, we hypothesize that this represents co-evolution between *P. leucopus* and *I. scapularis* larvae, which have shared the North American continent for many generations. An interesting future direction of this work is to examine how the tick bite changes over time, as it could be possible that the differences between *M. musculus* and *P. leucopus* become more substantive as feeding continues, although past work has demonstrated that the *P. leucopus* nymphal tick bite site becomes more inflamed over time (21). We do note that, as we have shown in Fig. S1, controlling for baseline skin gene expression in tick bite studies can be challenging, and so future studies may benefit from utilizing multiple controls when trying to understand these phenomena.

We consider two ways to interpret the QIAGEN IPA analysis, suggesting lower activation of proinflammatory cytokine signatures in *P. leucopus*: either *P. leucopus* spp. do not respond to *I. scapularis* tick bites or they respond to tick bites by upregulating homeostatic immune processes (reviewed in references [39, 40]). While we do not detect robust evidence of the latter in our RNA-sequencing data set, we note that subtle changes in anti-inflammatory factors, cellular metabolism (41), or tissue resident macrophages (42) could be difficult to detect by bulk RNA-sequencing despite having major impacts. This highlights the need for additional studies leveraging more sensitive tools, including single-cell RNA sequencing.

Regardless of whether larval ticks use stealth or recruitment of homeostatic processes to feed on competent hosts, there have been two non-mutually exclusive hypotheses proposed to describe host susceptibility (5). The first is that permissive hosts have immune systems which are intrinsically conducive/non-responsive to tick feeding. The second is that the tick is well suited through its salivary proteins to manipulate certain host responses locally. One interpretation of our data may be that saliva at different stages of tick development (larvae vs nymph) may impact host responses differently. This could be the result of different salivary protein content or saliva abundance, particularly given the small size of larval ticks and the low volume of their secreted saliva compared to nymphal ticks. Salivary components could further be impacted by differences in the tick responses to different blood sources. The composition of *I. scapularis* saliva has been found to change based on the host on which it is feeding, with *C. porcellus*, but not *M. musculus*, inducing salivary protein expression that, in turn, drives IL-4 production in murine splenocytes (43). Nevertheless, although nymphal ticks appear capable of circumventing innate immunity in *P. leucopus*, *M. musculus*, and humans, larvae appear to display some host specificity in their capacity to blunt the immune responses. We do note that while we see differences in inflammation, these data should not be misinterpreted: *I. scapularis* larvae can feed on all three hosts. It will be interesting to examine whether other wildlife species display differential immune responses to larval tick feeding and whether this contributes to feeding success. Additionally, in this study, BALB/c mice were used as representatives of *M. musculus* mice. However, additional studies may examine whether there are differences in *M. musculus* responses to larval ticks if different mouse strains and/or outbred mice are used.

These data add to a growing number of reports (44–46) that highlight issues with using *M. musculus* as a model of the North American *B. burgdorferi* enzootic cycle—the cycle through which the spirochete transitions between vertebrate and invertebrate hosts in nature (1). While *B. burgdorferi* can cycle through *M. musculus*—particularly in Europe—the predominant reservoirs in North America are *P. leucopus* and shrews (25, 47–49). The New World *Peromyscus* deer mouse is approximately 25 million years diverged from the Eurasian *Mus* mouse (50), meaning these species have undergone substantial independent evolution and that there has been ample opportunity for *I. scapularis* to coevolve with *P. leucopus*. While previous studies have focused on differences in how these rodents interact with *B. burgdorferi* (44–46), here we suggest that *P. leucopus* and *M. musculus* also have highly distinct cutaneous transcriptome responses to larval *I. scapularis* tick bites. Thus, while *M. musculus* has served as a very useful model for studying human Lyme disease severity and susceptibility, studies focused on the North American enzootic cycle should prioritize using natural reservoir species.

Past data from Europe examining human tick bite skin samples observed increases in neutrophils, B cells and CD8+, and tissue resident T cells, alongside small reductions in Langerhans cells, dermal dendritic cells, type-2 T cells (defined by IL-17A production), and numerous subsets of innate lymphoid cells (51). Curiously, additional impacts on human immune cell populations were observed in peripheral blood, including driving increased neutrophils but with reduced T cells and some innate lymphoid cell subsets. Together, these data point toward an activated, but nuanced, immune response to tick bites. This pairs with recent data showing a very small number of consistently differentially expressed genes in four patients with attached *I. scapularis* ticks (38). While these studies each differ in their methodologies and from the methodologies used in this work, we note that our study with larval *I. scapularis* showed robust immune activation, which the QIAGEN IPA analysis credited to interferon gamma, tumor necrosis factor, interleukin-1, and other cytokine signaling. Both our work, using the QIAGEN IPA software (Table S4), and the past study (38) identified evidence of enhanced IL-17 signaling at the transcriptomic level. It is therefore tempting to speculate that, similar to our observations with *M. musculus* (Fig. 3), larval ticks may induce more robust and canonically proinflammatory signaling when feeding on humans than nymphal stage ticks. Also, it should be noted that we used different sources of larval *I. scapularis* ticks for rodent and human placements. The contributions of natural genetic diversity across *I. scapularis* ticks to immune responses have not been reported and would be an area for future research.

Our data comparing hosts with good or bad tick feeding do not support an association between tick feeding success and proinflammatory transcriptomic responses in humans. However, there are several caveats to this observation. In the 4–6 days between tick placement and tick removal visits, subjects were asked to keep the dressing surrounding the ticks dry and not to wear strong-smelling perfumes or lotions. However, non-compliance with this guidance or other unrecognized variables could potentially have obscured differences in successful tick attachment and feeding. For example, differences in ingestion of garlic or B vitamins, which were not controlled for in this study, have anecdotally been reported to influence the ability for ticks to feed, although we are unaware of well-controlled studies regarding tick feeding and diet. Furthermore, our protocol for collecting the skin biopsies did not capture the participants with the worst feeding, as skin biopsies were only collected from subjects with at least one fed tick. Similarly, because inflammation was measured late into feeding, early transcriptomic differences that could have resulted in tick detachment would have been missed. Thus, future work could benefit from longitudinal data of human tick feeding.

In conclusion, these findings provide new insights into divergent host responses to larval *I. scapularis* tick feeding. Further studies might examine how this skin inflammation affects the tick-host-pathogen interface during enzootic cycling. Curiously, despite observing a stronger proinflammatory response here at the larval tick-*M. musculus* interface, past work demonstrates that *B. burgdorferi* is actually more readily transmitted from *M. musculus* to larval *I. scapularis* than from *P. leucopus* to *I. scapularis* (44, 46). Thus, the enhanced inflammation we observed at the *M. musculus* larval tick bite apparently does not impair transmission of the spirochete to new invertebrate hosts. It is possible that the opposite relationship could occur: the presence of *B. burgdorferi* in the skin could induce inflammation that could make it harder for *I. scapularis* to successfully feed on infected hosts. It would be interesting to observe whether this occurs, as well as whether there are differences in how *B. burgdorferi* impacts tick feeding across different vertebrate hosts.

## MATERIALS AND METHODS

### Rodent maintenance and use

Rodents were maintained by the Tufts Comparative Medicine Services. The *P. leucopus* colony was started by Dr. Sam Telford using wild-captured rodents from the northeastern and midwestern United States. The colony has been closed since 1994, held in microisolator cages, and is specifically pathogen free (confirmed by regular sentinel testing). BALB/c mice were obtained from Charles River Laboratories (Strain Code 028).

Equal numbers of male and female rodents were used at the beginning of the experiment. However, variable rates of successful recovery of ticks and/or RNA led to sex imbalance: two male and three female *P. leucopus* spp. were used in the RNA sequencing study. One male and three female BALB/c mice were used in the RNA sequencing study.

### Rodent tick infestation

A heated 1:4 mixture of melted beeswax-to-rosin gum mixture was used to attach a modified microcentrifuge tube lined with mesh between the shaved shoulder blades of each rodent. Ten *I. scapularis* larvae (National Tick Research and Education Resource, Oklahoma State University) were placed inside the mesh cap. Mice were singly housed in cages surrounded by a water moat for 48 hours. The tick containment chamber was carefully removed, and a 2 mm punch biopsy was taken surrounding a single feeding tick and transferred immediately to RNAlater, stored overnight at room temperature, and then frozen at –80°C until use. Biopsies were intentionally restricted to regions away from the edge of the tick containment chamber where damage from removal could skew results. A second biopsy was taken on the outside of the tick containment chamber as a matched control.

### Human subjects

The subjects described in this study were enrolled in the Xenodiagnosis After Antibiotic Treatment for Lyme Disease clinical study (NCT02446626). Participants were enrolled at Tufts University in Boston, Massachusetts; National Institutes of Health in Bethesda, Maryland; Mansfield Family Practice in Storrs, Connecticut; and Stony Brook University in Stony Brook, New York. Sequencing of banked biopsy samples was approved by the Tufts University Institutional Review Board.

### Human tick preparation, placement, and removal

Larval *Ixodes scapularis* ticks were reared at a central facility and provided to each of the testing centers. Tick placement and removal were performed as described by Turk et al. (52). For some participants in the post-treatment Lyme disease syndrome group, punch skin biopsies were taken at a control site prior to tick placement, and for all participants, biopsies were taken at the completion of tick feeding, at the site of a tick bite.

### Rodent RNA library preparation and RNA sequencing

After all rodent samples were collected as described above, samples were thawed on ice, transferred to QIAzol, and bead beaten with a 6.35 mm chrome steel bead (Biospec Products) for two cycles of 2 minutes each at an oscillation frequency of 30/second using a TissueLyser II (QIAGEN). RNA was extracted using the miRNeasy mini kit (QIAGEN), with the modification that two RWT washes were performed. gDNA was digested using TURBO DNase (Invitrogen), and RNA was repurified using the RNeasy MinElute Cleanup Kit (QIAGEN). Purified RNA was submitted to Azenta Life Sciences/Genewiz for library preparation and sequencing (Illumina HiSeq 2 × 150 bp).

### RT-qPCR

RNA was generated identically to the RNA sequencing experiment, with the exception that the Monarch Spin RNA Cleanup Kit (NEB) was used to repurify RNA after DNase treatment. RNA was subjected to cDNA synthesis using the ImProm-II Reverse transcriptase kit (Promega), and quantitative PCR was performed using the iTaq Universal SYBR Green Supermix (Bio-Rad). Reactions were performed on a Bio-Rad CFX Connect Real-Time system (one cycle of 95°C for 15 minutes, 40 cycles of 95°C for 30 seconds, 57°C for 30 seconds, and 72°C for 30 seconds, and one cycle of 95°C for 1 minute and 55°C for 1 minute). Primers are listed in Table 2. All data are presented as relative to unbitten skin outside of the tick placement cap (e.g., a gene with a negative log_2_fold change has reduced expression under the cap compared to outside of the cap). Primer efficiencies were calculated based on a twofold dilution series.

**TABLE 2:** Primers used in this study

Lab designation	Target	Sequence	Efficiency (%)	Citation
JSB_Pr13	*P. leucopus 18s* Forward	GCTCCTCTCCTACTTGGATAAC	101	This work
JSB_Pr14	*P. leucopus 18s* Reverse	CTGATAAATGCACGCATCCC		This work
JSB_Pr27	*M. musculus 18s* Forward	GCAATTATTCCCCATGAACG	97	(53)
JSB_Pr28	*M. musculus 18s* Reverse	GGGACTTAATCAACGCAAGC		(53)
JSB_Pr126	*P. leucopus Fosb* Forward	CAACCCACCCTCATCTCTTC	96	This work
JSB_Pr127	*P. leucopus Fosb* Reverse	CTGGTTCCTGGCATGTCATA		This work
JSB_Pr128	*M. musculus Fosb* Forward	AGGAACAAGGAGGAGGAAGA	98	This work
JSB_Pr129	*M. musculus Fosb* Reverse	GGTCAGACAGAAGAGTCAAAGG		This work
JSB_Pr134	*P. leucopus Ccr7* Forward	GGTTCCTCCCTCTCATGTATTC	107	This work
JSB_Pr135	*P. leucopus Ccr7* Reverse	GTCATGGTCTTGAGCCTCTT		This work
JSB_Pr136	*M. musculus Ccr7* Forward	GCTCAAGACCATGACGGATAC	96	This work
JSB_Pr137	*M. musculus Ccr7* Reverse	GGCCCAGAAGGGAAGAATTAG		This work
JSB_Pr138	*P. leucopus ccl5* Forward	GCCCACGTCAAGGAGTATTT	99	This work
JSB_Pr139	*P. leucopus ccl5* Reverse	TCCTGAACCCACTTCTTCTTTG		This work
JSB_Pr151	*M. musculus Ccl5* Forward	CACCATATGGCTCGGACACC	100	This work
JSSB_Pr152	*M. musculus Ccl5* Reverse	TCTGGGTTGGCACACACTTG		This work

### Human RNA library preparation and RNA sequencing

Skin biopsies (2 mm) from subjects were stored in RNAlater overnight at room temperature and then frozen at –80°C until use. Samples were homogenized under liquid nitrogen using a mortar and pestle. Tissues were then resuspended in 1 mL of lysis buffer from the RNeasy Fibrous Tissue Mini Kit (QIAGEN) and processed as per the manufacturer’s protocol. Ribosomal RNA was depleted, and RNA-seq libraries were prepared by Tufts University Core Facility. Sequencing of 50 bp single-end reads was performed on a HiSeq 2500.

### RNA-seq analysis

Reads were mapped to *P. leucopus* (GCF_004664715.2) (54), *M. musculus* (GRCm39), or human genomes (GRCh38.98) using STAR (version 2.6.1) (55), and gene expression was summarized using RSEM (version 1.3.1) (56). Principal component analysis was performed using the R Bioconductor PCAtools package (R version 4.3.1) (57). Differential expression analysis was performed using DESeq2 (R version 4.3.1) (58). Genes with low expression were removed based on their normalized read counts. Genes with greater than 10 read counts were kept. Differentially expressed genes were identified using an adjusted *P* value cutoff of 0.05. Genes with no expression and T-cell receptor genes were removed prior to plotting. Heatmaps and clustering, based on Euclidean distance, were generated using the ComplexHeatmap package (R version 4.3.1) (59). Pathway analysis was performed with the use of QIAGEN Ingenuity Pathway Analysis (QIAGEN Inc., https://digitalinsights.qiagen.com/IPA) (36). IPA used differentially expressed genes from the DESeq2 differential expression analysis with *P*_adj_ ≤0.05.

## Data Availability

Rodent sequencing data are deposited in GEO (GSE266088), and data from the human clinical trial are available in dbGaP (phs003314.v1.p1).

## SUPPLEMENTAL MATERIAL

Fig. S1 iai.00065-25-s0001.tif fosb induction as a function of tick feeding.

Supplemental material iai.00065-25-s0002.docx Legends for supplemental figure and tables.

Table S1 iai.00065-25-s0003.xlsx DESeq2 analysis of BALB/c *M. musculus* and *P. leucopus* gene expression following larval tick bite.

Table S2 iai.00065-25-s0004.xlsx QIAGEN IPA analysis of BALB/c *M. musculus* and *P. leucopus* signaling following larval tick bite.

Table S3 iai.00065-25-s0005.xlsx DESeq2 analysis of human gene expression following larval tick bite. Data contain two analyses: pre- and post-tick placement and good (>20 fed ticks) vs bad (<10 fed ticks) feeding.

Table S4 iai.00065-25-s0006.xlsx QIAGEN IPA analysis of human signaling following larval tick bite.

ASM does not own the copyrights to Supplemental Material that may be linked to, or accessed through, an article. The authors have granted ASM a non-exclusive, world-wide license to publish the Supplemental Material files. Please contact the corresponding author directly for reuse.
