# Supplementary figures and images for: Peromyscus leucopus, Mus musculus, and humans have distinct transcriptomic responses to larval Ixodes scapularis bites
Source: Infect Immun. 2025 Mar 11;93(4):e00065-25. doi: 10.1128/iai.00065-25 (PMC11977304; doi:10.1128/iai.00065-25)

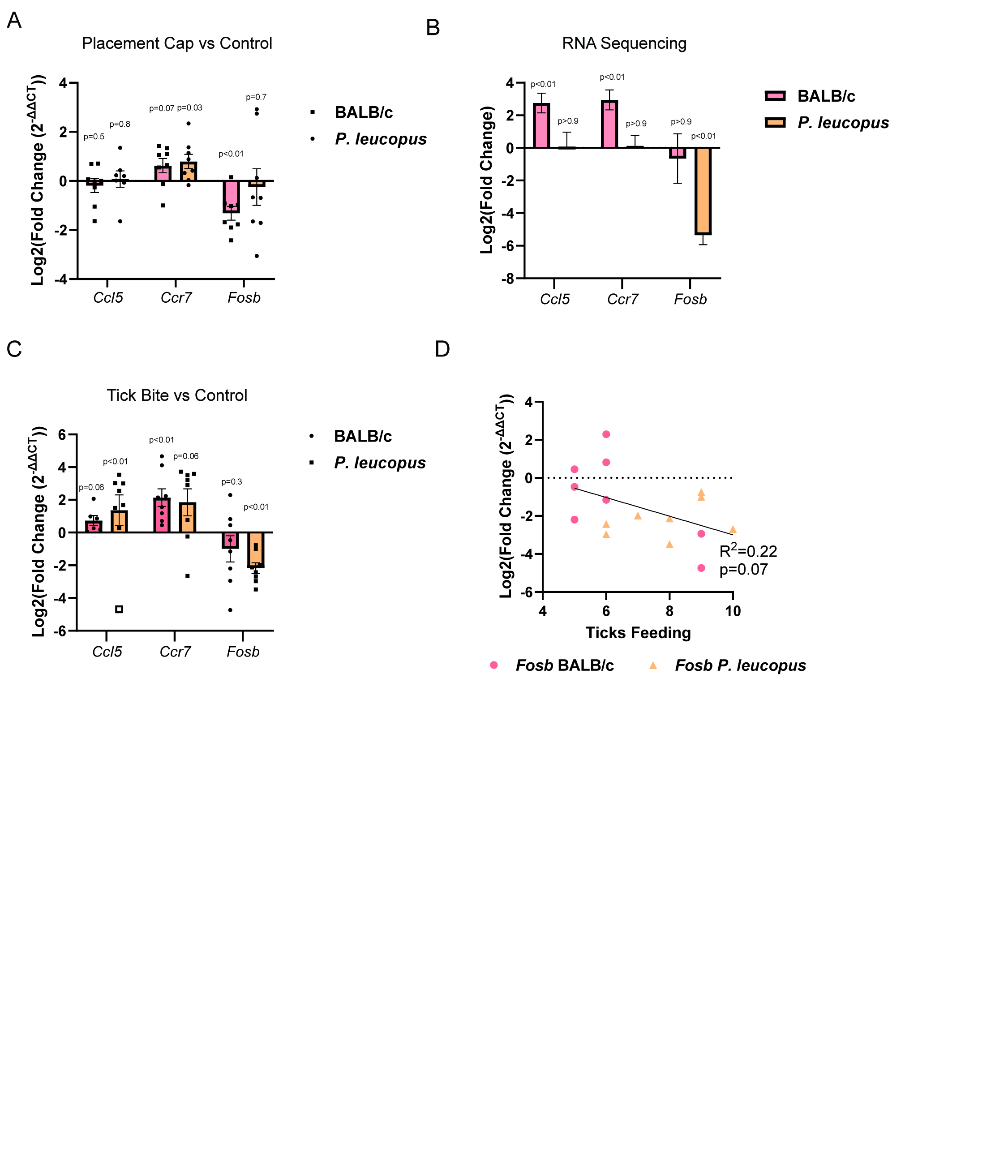

Supplement: Fig. S1 — fosb induction as a function of tick feeding. [file iai.00065-25-s0001.tif]
