# Supplementary material for: Peromyscus leucopus, Mus musculus, and humans have distinct transcriptomic responses to larval Ixodes scapularis bites
Source: Infect Immun. 2025 Mar 11;93(4):e00065-25. doi: 10.1128/iai.00065-25 (PMC11977304; doi:10.1128/iai.00065-25)
Supplement: Supplemental material — Legends for supplemental figure and tables. [file iai.00065-25-s0002.docx]

Supplemental Materials Legends

Table S1: DESeq2 analysis of BALB/c *M. musculus* and *P. leucopus* gene expression following larval tick bite.

Table S2: QIAGEN IPA analysis of BALB/c *M. musculus* and *P. leucopus* signaling following larval tick bite.

Table S3: DESeq2 analysis of human gene expression following larval tick bite. Data contain two analyses: Pre- and Post-tick placement and good (>20 fed ticks) vs bad (<10 fed ticks) feeding.

Table S4: QIAGEN IPA analysis of human signaling following larval tick bite.

**Figure S1: Examining *fosb* induction as a function of tick feeding**. (A) RT-qPCR analysis of *Ccl5*, *Ccr7*, and *Fosb* differential gene expression in response to the tick placement chamber. Each dot represents a single punch biopsy from under a placement cap. Two biopsies were taken per rodent and normalized to one biopsy from outside of the cap. (B) RNA sequencing data for *Ccl5*, *Ccr7*, and *Fosb*. P-values are based on an FDR correction. Data from **Figure 1, Table S1**. (C) RT-qPCR analysis of *Ccl5*, *Ccr7*, and *Fosb* expression in response to larval tick bites. Each dot represents an individual rodent. For A and C, transcripts were normalized to 18s ribosomal RNA and fold change was calculated as 2^-ΔΔCT^. P-values calculated from a one-sample T-test compared to 0. (D) There is not a correlation between successful tick feeding and *Fosb* expression. P-value and R^2^ values calculated from simple linear regression. Data for A-D are derived from two independent experiments. Male and female mice were used in each experiment.
